# Assessment of the Functional Status of Patients After Stroke Depending on the Length of Stay in the Rehabilitation Ward—A Retrospective Single-Center Study

**DOI:** 10.3390/jcm15093306

**Published:** 2026-04-26

**Authors:** Michał Małek, Anna Hozakowska, Zbigniew Guzek, Małgorzata Stefańska, Joanna Kowalska

**Affiliations:** 1 Faculty of Physiotherapy, Wroclaw University of Health and Sport Sciences, 50-367 Wrocław, Poland; m.malek@inz.uz.zgora.pl (M.M.); malgorzata.stefanska@awf.wroc.pl (M.S.); 2 Department of Physiotherapy, University of Zielona Góra, 65-046 Zielona Góra, Poland; a.hozakowska@inz.uz.zgora.pl; 3 Department of Neurological Rehabilitation, University Hospital in Zielona Góra, 65-046 Zielona Góra, Poland; guzek@o2.pl

**Keywords:** stroke, functional status, time after stroke, post-stroke rehabilitation, length of stay, effectiveness of rehabilitation

## Abstract

**Background/Objectives:** Swift achievement of an optimal functional status after stroke may substantially reduce patients’ stay in a medical facility and enable their return home. The study aimed to assess the functional status in patients after their first stroke, depending on the time after stroke incident and the patient’s length of stay in the stroke rehabilitation ward. **Methods**: The data from 229 patients, aged 69.4 ± 11.3 years (120 men and 109 women), formed part of the analysis. Based on medical records, basic socio-demographic, clinical data, and the results of the tests—Mini Mental State Examination (MMSE), Barthel Index (BI), Time Walk Test 10 m (TWT), Trunk Control Test (TCT), Up and Go Test (TUG), and Berg Balance Scale (BBS)—were collected. **Results**: The risk groups for patients with longer post-stroke rehabilitation stays and poorer rehabilitation outcomes included women, older adults, those with poorer functional status, and patients admitted to the stroke rehabilitation ward after a longer period of time after stroke. **Conclusions**: The functional status and the length of stay in the post-stroke rehabilitation ward should be monitored and analyzed to find and support groups of patients who may rehabilitate more slowly and stay longer in the ward. A shorter patient stay can allow for more effective management of beds in the post-stroke rehabilitation wards.

## 1. Introduction

Stroke remains the leading cause of disability among adults and the second most frequent cause of death [1,2]. It pertains to a number of disorders at the structural, functional, cognitive–emotional, and social levels.

Stroke imposes a growing financial burden on patients, families, health systems, and economies worldwide. The estimated global cost of stroke is over US $ 890 billion (0.66% of the global gross domestic product, GDP) per year and is projected to almost double by 2050 [3].

Nearly 90,000 stroke cases are reported every year in Poland, of which 30% occur in persons below the age of 65 [2].

The increased incidence may be attributed to several different factors which encompass 23 modifiable factors [1]. Thanks to treatment in the neurology ward, among others, in the form of thrombolytic therapy or mechanical thrombectomy in cases of ischemic strokes and surgical treatment in cases of hemorrhagic strokes, continuing treatment in the rehabilitation ward is of utmost significance [4].

The diverse pathogenesis and variety of stroke symptoms require a holistic approach to each patient and a comprehensive therapy, implemented already at the level of the neurology ward and subsequently followed by the post-stroke rehabilitation [5]. The primary goal is to help regain independent functioning. Appropriate rehabilitation, tailored to the patient’s individual needs and application of modern neuro-rehabilitation methods, provides patients with the opportunity to regain autonomy in carrying out daily activities, as well as pursue their passions or return to professional activity [6].

In Poland, rehabilitation of stroke patients is provided by the private sector or is contracted by the National Health Fund (NFZ). The length of stay in early neurological rehabilitation wards directly after the stroke ranges from 6 to 16 weeks (42–112 days) and is similar to the duration of stay in other countries [7,8]. Secondary rehabilitation in systemic rehabilitation wards tends to be realized in subsequent months and lasts between 3 and 6 weeks at a time [9,10].

Meanwhile, contracting post-stroke rehabilitation worldwide involves two models: public and private. The world public sector, similar to the one in Poland, is financed by health insurance and carried out by various public medical facilities. Western European countries follow this particular approach. The private model, where the patient pays for the treatment themselves or avails of private insurance, is a more popular solution, for instance, in the USA [1,11].

However, reports indicate insufficient access to post-stroke rehabilitation. Only 22% of patients begin rehabilitation within the crucial two-week period following the incident [2].

Hence, it is essential to strive to achieve the highest possible effectiveness of rehabilitation in stroke rehabilitation wards; thus, an improved functional status and a regained independence in the shortest possible period of time. This is a key objective for proper capacity management in rehabilitation facilities.

Swift achievement of an optimal functional status may substantially reduce patients’ stay in a medical facility and enable them to return home. These matters are of crucial importance both in terms of costs incurred by the healthcare system and the quality of life of stroke survivors, as well as a shorter waiting time for admission to a medical facility [12].

Therefore, the aim of this study was to assess the functional status in patients after their first stroke, depending on the time elapsed since the stroke incident and the patient’s length of stay in the stroke rehabilitation ward.

Such studies are important not only from a scientific perspective but also from an application point of view. They may constitute a basis for increasing the capacity of stroke wards as the primary level of care, as well as advancing the percentage of patients obtaining effective care in a short time perspective, and thus, preventing lifelong disability and ensuring functional independence after stroke [1].

## 2. Materials and Methods

### 2.1. Studied Group

This retrospective observational study was conducted at the Neurological Rehabilitation Ward of the Department of Rehabilitation of the University Hospital in Zielona Góra, Poland, and was approved by the Ethics Committee of the Health and Sport Sciences in Wroclaw, Poland (reference no 23/2024). Additionally, the study was conducted in accordance with the Helsinki Declaration.

Relevant data on the subject were collected from the medical records of stroke patients hospitalized between January 2022 and June 2025. The analysis encompassed data concerning 318 patients.

**Figure 1:**
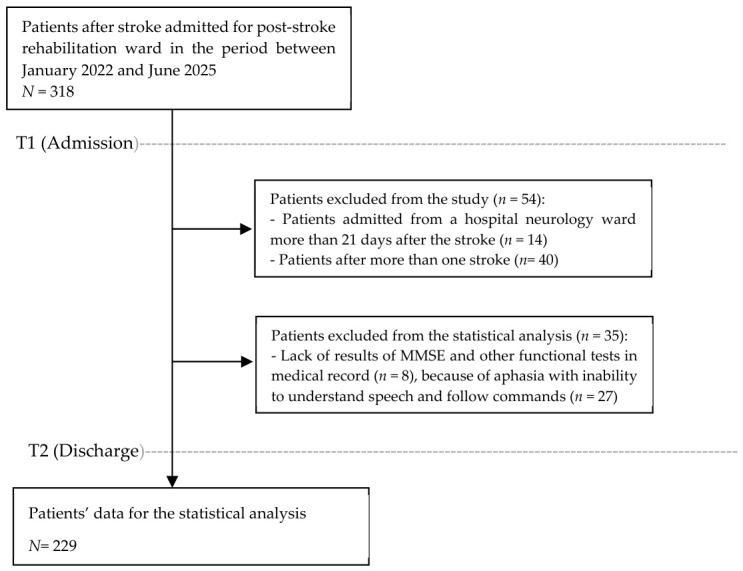
The data recruitment process for statistical analysis.

The following inclusion criteria were adopted: first stroke, assessment of cognitive function using the Mini Mental State Examination (MMSE), and patients admitted from a hospital neurology ward. Exclusion criteria included: aphasia with inability to understand speech and follow commands; missing results of standard functional and cognitive assessment tests; presence of serious mental disorders in the medical records, e.g., altered consciousness or depression; and admission to a post-stroke rehabilitation ward more than 21 days after the stroke (Figure 1).

Ultimately, data from 229 patients meeting the inclusion criteria, aged 69.4 ± 11.3 years, including 120 men and 109 women, formed part of the analysis. The majority of the studied group consisted of patients with ischemic stroke and right hemisphere involvement. Detailed data have been presented in Table 1.

### 2.2. Measurement Tools

Based on medical records, basic socio-demographic and clinical data were collected, along with the results of the following tests: Mini Mental State Examination (MMSE), Barhtel Index (BI), Time Walk Test 10 m (TWT), Trunk Control Test (TCT), Up and Go Test (TUG), and Berg Balance Scale (BBS).

MMSE is a short mental state scale that comprises 30 questions and instructions assessing such functions as orientation in time, orientation in place, memory, attention and calculation, recall, naming, repetition, comprehension, reading, writing, and drawing. The participant receives 1 point for each correct answer. Each participant can receive a maximum of 30 points, with values ranging from 0 to 10 points indicating suspected severe dementia, 11–18 indicating suspected moderate dementia, 19–23 indicating suspected mild dementia, 24–26 indicating mild cognitive impairment without dementia, and 27–30 indicating a normal score [13,14].

BI is designated to assess the functional status based on ten basic activities of daily living. The modified Barthel scale allows the participant to score a maximum of 20 points, with higher scores indicating better functional status and, consequently, greater ability to function independently. Values 0–7 indicate severe functional status, 8–15 show moderate status, while 16–20 indicate mild status [15].

TWT (10 m) assesses patients’ walking speed over a distance of 10 m. It involves covering a distance of 10 m in the shortest possible time. The higher the walking speed, the shorter the walking time and, simultaneously, the better the walking ability [16].

TCT is used to assess patient mobility and consists of four tasks: turning to the affected side, turning to the unaffected side, moving from a lying to a sitting position, and sitting with legs lowered. The lack of ability to perform the task without assistance is awarded 0 points, the ability to perform movement with partial assistance is awarded 12 points, and independence in performing the task is awarded 25 points. The participant may receive a maximum total of 100 points [17].

TUG is used to assess such features as balance, postural control, and walking. The test involves measuring the walking time from standing up from a chair, walking 3 m, turning around, and sitting back down. Three attempts are allowed, with the shortest time being considered. The patient may use walking aids, such as a walker or cane, in the course of the test. The shorter the time, the lower the risk of falling. Values between 20 and 29 s require supervised walking, whereas values above 30 s require assistance during movement due to the very high risk of falling [18].

BBS determines the participant’s ability to maintain balance while performing specific tasks. The scale comprises 14 movement tasks related to static and dynamic balance. For each task, the participant may obtain from 0 to 4 points. The maximum total score amounts to 56 points. The higher the total score, the better the balance. Values of 0–20 indicate a high risk of falls, 21–40 show a moderate risk of falls, whilst 41–56 indicate a low risk of falls [19].

These tests are routinely performed upon admission to the stroke rehabilitation ward (T1) and upon discharge from it (T2). Patients received regular physiotherapy from Monday to Friday, twice a day for 60 min, and once a day on Saturdays for 40 min. The physiotherapy program was individually tailored to the patient’s functional status and included individual work with a physiotherapist, learning and/or improving gait on flat terrain and up and down the stairs.

### 2.3. Statistical Analysis

The ordinal nature of the majority of the analyzed variables contributed to the use of the median as a measure of central tendency and the interquartile range (IQR-Q3-Q1) as a measure of dispersion. In case of nominal variables, frequencies and percentages were calculated. The Wilcoxon signed-rank test was applied in order to determine the significance of differences between two dependent variables. Kruskal–Wallis ANOVA was applied in order to determine the significance of differences between multiple independent variables, and when appropriate, Dunn Bonferroni–Holm post hoc tests were used.

The effect size for the Wilcoxon signed-rank test was estimated using the r coefficient according to the formula r = Z/$\sqrt{n}$ [20]. The effect size for the Kruskal–Wallis ANOVA was estimated using the Eta squared (*η*^2^) value transformed to Cohen’s d coefficient [21]. Statistical significance was determined when *p* < 0.05. Analyses were conducted with Statistica 14.1 and PQStat 1.8.4 software.

## 3. Results

In the studied group of post-stroke patients, a statistically significant change was demonstrated between T1 and T2 scores. Significant improvements were observed in such features as cognitive and functional status, balance, mobility, and locomotion (Table 2).

Taking into account the time elapsed from the stroke incident to admission to the post-stroke rehabilitation ward, patients were divided into three groups: up to 8 days from the stroke, 9–14 days from the stroke, and 15–21 days from the stroke to admission to the rehabilitation ward. A statistically significant difference was observed between the groups in BI, TWT, and TUG in T1 (Table 3). At the time of discharge from the ward (T2), no material differences were observed in the functional status and mobility of the subjects. Patients with the longest time from stroke to admission to the rehabilitation ward stayed in the ward the longest, although these differences were not statistically significant (Table 3).

Considering the length of stay in the ward, patients were divided into five groups (up to 3 weeks, up to 6 weeks, up to 9 weeks, up to 12 weeks, and over 12 weeks). Statistically significant differences were identified in BI, TWT, TCT, TUG, and BBS between the analyzed groups in T1. However, in T2, a statistically significant difference was noted only in BBS (Table 4).

No significant differences were identified in the time of admission to the rehabilitation ward or the length of stay in the ward depending on the type of stroke and the affected hemisphere, gender, or marital status of the participants (*p* > 0.05).

The statistically significant multivariate regression model demonstrated that gender, age, and MMSE in T1, and length of stay in the ward had a significant impact on BI in T2. In accordance with the model, higher BI scores in T2 were observed in men. A positive effect of MMSE in T1 was also demonstrated: a 5-point higher MMSE score predicted a 2.1-point higher BI score, and a negative effect of age and length of stay in the hospital (lower values of the dependent variables predict higher values of the dependent variable) (Table 5).

## 4. Discussion

The process of rehabilitation is essential in the treatment of stroke patients. Improving functional ability and mobility are fundamental to achieving independence in one’s daily life. The presented study assessed the functional status of the participants and its change depending on the time elapsed from the stroke to admission to the post-stroke rehabilitation ward, as well as the duration of the patients’ stay in that ward.

The study demonstrated that all patients, regardless of the above-stated two parameters, achieved improvements in their functional status, mobility, and locomotion. This is consistent with previous reports, as many authors have observed improvements in these parameters after the post-stroke rehabilitation period, regardless of its stage [22,23,24].

The results of detailed analyses revealed that the functional status of the participants, their mobility, and gait varied depending on the time elapsed from the stroke to admission to the rehabilitation ward. Patients admitted to the rehabilitation ward between 15 and 21 days after stroke had significantly poorer functional status (BI) and significantly lower scores on mobility and gait tests (TWT and TUG). This may have been linked to the patient’s poorer overall clinical condition, the extent of neurological sequelae, the course of the acute phase of the stroke, and the treatment regimen during hospitalization in the neurology ward. This group also had the longest length of stay in the ward. This complies with the results of the study by Bijl et al., whereby a one-point score increase on the FIM scale (motor assessment of mobility) on admission shortened the length of stay by almost one-third of a day [25].

Otokita et al. also pointed out that earlier rehabilitation typically resulted in shorter lengths of stay and a higher BI [26]. He et al. also indicated in their study that patients who did not start hospital rehabilitation within 24 h after being considered clinically ready had slower and worse recoveries and longer stays in rehabilitation [27]. Furthermore, the authors pointed out yet another important issue. The main reason for delaying the start of rehabilitation was not due to medical reasons but rather a lack of available beds/spaces in the rehabilitation ward (this concerned over 96% of cases) [27].

Nevertheless, the results recorded at the time of patient discharge demonstrated improved functional status, mobility, and gait, and a reduction in significant differences observed at the time of admission, regardless of the time since the stroke. Coleman et al. also concluded in their narrative review that the optimal time to start rehabilitation after stroke remains unknown, though increasing evidence indicates that, for at least some deficits, implementing rehabilitation strategies within the first two weeks after stroke is beneficial [28].

Yet another analysis performed depending on the duration of stay in the ward revealed that the longest stay (over 12 weeks) in the ward was observed in patients who were admitted in the worst functional condition and had significant problems with balance, movement, and locomotion, as well as basic activities of daily living compared to the other groups. This was also the largest group of patients.

In this case, too, at the time of discharge from the ward, an improvement in the parameters studied was observed. Their functional status remained poorer compared to the other groups; however, the differences were not statistically significant. Balance remained the most problematic of all issues.

Similar results were reported by Cinciu et al., in which patients with a higher degree of disability required a longer hospitalization period [12]. However, the studies carried out by Bindawas et al. showed that short (≤30 days) and intermediate (31–60 days) length of stays were associated with higher functional outcomes at discharge [29].

Thus, it would seem that early post-stroke rehabilitation, with its systemically defined duration of up to 16 weeks after stroke in Poland, is justified and should be the standard in the treatment and rehabilitation of stroke patients. However, effective rehabilitation assumes improvement in the patient’s condition in the shortest possible time. Therefore, patients who demonstrate a faster rate of improvement achieve therapeutic goals earlier and are therefore discharged from the ward more quickly, while patients with poorer functional status and/or stroke-related complications (but also those who improve functionally) remain in the rehabilitation ward longer, which may indicate a seemingly greater effectiveness of rehabilitation during a longer stay [29,30].

Moreover, James and McGlinchey noted in their study that patients with more severe stroke engaged in less active exercise during rehabilitation, which may be related to a longer stay in the stroke ward [31].

Our results suggest that the duration of stay of 4–6 or 7–9 weeks, but no longer than 12 weeks, was optimal for improving the studied parameters in analyzed group of patients. Regression analysis also shows a correlation between these two parameters. However, it does not mean that shorter stays improve functional outcomes. The length of stay is itself a downstream consequence of recovery trajectory, case complexity, and discharge readiness. It only means, especially in our observational study, that patients with better functional status were discharged earlier. Previous studies have emphasized that it is the early initiation of rehabilitation and its intensity, not the length of stay, that translates into its effectiveness and functional improvement for the patient [26]. Discharging patients from the neurology ward and redirecting them to the post-stroke rehabilitation ward for early rehabilitation can reduce the social burden associated with stroke [32,33].

Regression model demonstrated that, among the analyzed factors, younger age, male gender, and better cognitive status were associated with greater improvement in functional status and greater independence in the studied stroke patients at discharge. It should be noted that the regression model explains this association only moderately. Further studies and analyses are needed in multicenter settings and with larger patient populations. Other researchers have had similar observations [29,34]. Older patients demonstrate higher levels of disability, and, as Canciu et al. reported, the disability score increases with each passing year of life [12]. Furthermore, many studies reported poorer functional recovery after stroke for women compared to men [35,36]. Researchers have also agreed and emphasized that cognitive function is the predictor of functional status in stroke patients [17,37].

The results are thought-provoking and suggest that there is a group of patients after stroke in whom the improvement in functional status may take longer, which may therefore extend the stay in the post-stroke rehabilitation ward. Therefore, this could be a group of patients who require additional support to improve their functional status in a shorter time during the ward’s stay.

In this respect, rehabilitation programs can be supplemented with interventions that support cognitive function, as well as the assessment and optimization of cognitive function, during rehabilitation [38]. Furthermore, physiotherapists ought to carefully tailor their sessions, especially for patients with severe stroke, to maximize the amount of activity undertaken [31].

Such observational analyses (as the one presented in this article), in addition to those conducted in various centers, can be valuable not only to researchers but also to clinicians and administrators of rehabilitation centers, who are striving to more effectively manage bed occupancy and human resources in post-stroke rehabilitation.

However, this requires expansion and continuation of research.

The studies presented above have certain limitations. First and foremost, these are observational single-center studies, so caution should be exercised in generalizing the results. The studies do not consider all factors that could be associated with functional status. The analysis was based on data collected from medical records. The type of treatment administered during hospitalization and the affected brain areas (which may result in, for example, spasticity, hemineglect, or visual impairment) were not considered, only the affected cerebral hemisphere. Adjustment for spontaneous recovery, or a clearer account of discharge criteria, was not taken into account. Additionally, patients with aphasia, those who did not understand instructions, those with missing MMSE scores, patients admitted more than 21 days after stroke, and those with multiple strokes were excluded from the analysis. This group limitation may have been related to the results obtained and the possibility of concluding. It is also unknown what the cognitive status was before the stroke. The cohort was dominated by ischemic stroke patients.

Nevertheless, the study provides a realistic picture of patients admitted to the post-stroke rehabilitation ward over a period of more than three years, which, in our opinion, has significant practical implications.

## 5. Conclusions

The analyzed group of patients noted improvements in functional status, mobility, and locomotion, indicating the effectiveness of the rehabilitation.

Patients admitted to the post-stroke rehabilitation ward between 15 and 21 days after their stroke had significantly poorer functional status, and scored significantly lower on tests assessing mobility and gait at the time of admission to the ward (T1) compared to patients with a shorter time elapsed since their stroke.

The largest group of patients, those staying in the ward for more than 12 weeks, had significantly poorer functional status and problems with balance and mobility at the time of admission to the ward (T1) compared to the groups with shorter post-stroke rehabilitation stays.

The functional status and the length of stay in the post-stroke rehabilitation ward should be monitored and analyzed to find and support groups of patients who may rehabilitate slower and stay longer in the ward.

## Figures and Tables

**Table 1:** Characteristics of the studied group (*N* = 229).

		*N*	%
Gender	Men	120	52.4
	Women	109	47.6
Marital status	In a relationship	109	47.6
	Single	120	52.4
Place of residence	City	168	73.4
	Village	61	26.6
Type of stroke	Ischemic	206	90.0
	Hemorrhagic	23	10.0
Affected hemisphere	Right	131	57.2
	Left	98	42.8
Comorbidities (number)	1	6	2.6
	2	163	71.2
	3	22	9.6
	≥4	38	16.6
		Mean	SD
Age		69.4	11.3
Length of stay in the rehabilitation ward [days]		62.5	34.5
Time from stroke to admission to the rehabilitation ward [days]		11.0	3.3

**Table 2:** The studied parameters in T1 and T2.

	T1		T2		Wilcoxon’s Test *p*	Effect Size r
	Medin	IQR	Medin	IQR		
MMSE	22	7	24	7	<0.0001 *	0.48
BI	6	8	16	9	<0.0001 *	0.87
TWT [s]	0	10	10	13	<0.0001 *	0.44
TWT speed [m/s]	0	0.67	0.71	1	<0.0001 *	0.85
TCT	100	52	100	0	<0.0001 *	0.76
TUG	0	12	10	15	<0.0001 *	0.41
BBS	23	31	46	26	<0.0001 *	0.82

T1—measurement 1; T2—measurement 2; BI—Barthel Index; MMSE—Mini Mental State Examination; TWT—Time Walking Test; TCT—Test Trunk Control; TUG—Up and Go Test; BBS—Berg Balance Scale; IQR—interquartile range; * *p* < 0.05.

**Table 3:** The examined parameters in T1 and T2, depending on the time from stroke to admission to the post-stroke rehabilitation ward.

Time from Stroke to Admission to the Post-Stroke Rehabilitation Ward								
	Up to 8 Days *n* = 65		9–14 Days *n* = 133		15–21 Days *n* = 31		ANOVA Kruskal–Wallis *p*	Cohen’s d
Results in T1	Median	IQR	Median	IQR	Median	IQR		
MMSE	22	6	22	5	20	9.5	0.3520	0.04
BI	6	8	7	9	3	6	0.0120 *	0.35
TWT [s]	0	10	0	11	0	0	0.0471 *	0.27
TWT speed [m/s]	0	0.66	0	0.67	0	0	0.0208 *	0.32
TCT	100	39	100	52	74	52	0.4267	0.07
TUG	0	10	0	13	0	0	0.0297 *	0.30
BBS	25	28	24	31	11	24.5	0.0688	0.25
**Results in T2**								
MMSE	23	8	24	7	23	12	0.5125	0.13
BI	17	6	17	9	15	9	0.1986	0.15
TWT [s]	10	8	9	13	10	13	0.7031	0.15
TWT speed [m/s]	0.71	0.69	0.76	1	0.67	0.83	0.1328	0.18
TCT	100	0	100	0	100	0	0.8904	0.18
TUG	12	9	10	15	13	16	0.6857	0.15
BBS	48	22	47	24	42	33	0.6600	0.25
Length of stay in the ward [days]	56	66	53	66	77	50	0.1556	0.17

T1—measurement 1; T2—measurement 2; BI—Barthel Index; MMSE—Mini Mental State Examination; TWT—Time Walking Test; TCT—Test Trunk Control; TUG—Up and Go Test; BBS—Berg Balance Scale; IQR—interquartile range; * *p* < 0.05.

**Table 4:** Parameters examined in T1 and T2, depending on the length of stay in the post-stroke rehabilitation ward.

	Length of Stay in the Post-Stroke Rehabilitation Ward										ANOVA	Cohen’s
	Up to 3 Weeks		4–6 Weeks		7–9 Weeks		10–12 Weeks		>12 Weeks		Kruskal -Wallis	
	*n* = 29		*n* = 58		*n* = 34		*n* = 32		*n* = 76			
Results in T1	Median	IQR	Median	IQR	Median	IQR	Median	IQR	Median	IQR	*p*	d
MMSE	23	7	23	6	22	8	20	7	21.5	9.5	0.1296	0.24
BI	10	9	8	8	8.5	5	4	6.5	3	6	<0.0001 *	0.73
TWT [s]	8	10	9	15	0	12	0	4	0	0	0.0003 *	0.57
TWT speed [m/s]	0.59	1	0.39	0.83	0	0.67	0	0.28	0	0	<0.0001 *	0.70
TCT	100	13	100	26	100	39	61.5	70	61	52	0.0001 *	0.61
TUG	9	10	10	15	0	12	0	5	0	0	0.0005 *	0.56
BBS	37	36	33	21	28	28	12.5	20.5	9	22.5	<0.0001 *	0.96
Time from stroke to admission to ward [days]	10	3	10	4	10	5	11	6	10	5	0.7186	0.19
**Results in T2**												
MMSE	24	6	24	8	23.5	7	23	12.5	24	7.5	0.4585	0.08
BI	17	8	18	5	16.5	4	14.5	12.5	15	10	0.0823	0.15
TWT [s]	8	6	10	5	10	6	9	12.5	9.5	14.5	0.3799	0.06
TWT speed [m/s]	0.83	1.07	0.83	0.45	0.83	0.42	0.61	1	0.51	1	0.0524	0.31
TCT	100	0	100	0	100	0	100	45	100	13	0.0551	0.31
TUG	8	12	10	6	12	7	10	15	11	16.5	0.2672	0.15
BBS	47	22	49	16	50.5	14	41.5	41.5	42	34	0.0043 *	0.46

T1—measurement 1; T2—measurement 2; BI—Barthel Index; MMSE—Mini Mental State Examination; TWT—Time Walking Test; TCT—Test Trunk Control; TUG—Up and Go Test; BBS—Berg Balance Scale; IQR—interquartile range; * *p* < 0.05.

**Table 5:** Predictors of functional status (BI) in T2.

Dependent Variable: BI in T2	Coefficient b	Error b	−95% CI	+95% CI	*p*
Gender (1—women, 2—men)	1.33	0.67	0.01	2.65	0.0476 *
Age	−0.08	0.03	−0.14	−0.02	0.0074 *
Type of stroke (1—ischemic, 2—hemorrhagic)	1.35	1.12	−0.86	3.56	0.2304
Affected cerebral hemisphere (1—left, 2—right)	−0.63	0.69	−1.99	0.72	0.3565
MMSE in T1	0.42	0.06	0.31	0.53	<0.0001 *
Time from stroke to admission to rehabilitation ward [days]	−0.16	0.10	−0.35	0.04	0.1218
Length of stay in the rehabilitation ward [days]	−0.02	0.01	−0.04	0.00	0.0270 *
Sample size assumption: n ≥ 50 + 8k	Yes				
*p*	<0.001				
Standard error of estimation	4.97				
R^2^ corrected	0.30				

T1—measurement 1; T2—measurement 2; BI—Barthel Index; MMSE—Mini Mental State Examination; CI- Confidence Interval; * *p* < 0.05.

## Data Availability

The data presented in this study are available from the corresponding author upon request.

## Abbreviations

The following abbreviations are used in this manuscript:

MMSE	Mini Mental State Examination
BI	Barthel Index
TWT	Time Walk Test
TUG	Up and Go Test
TCT	Trunk Control Test
BBS	Berg Balance Scale
T1	Measurement 1
T2	Measurement 2
IQR	Interquartile Range

## References

[1] Feigin V.L., Brainin M., Norrving B., Martins S.O., Pandian J., Lindsay P., F Grupper M., Rautalin I. (2025). World Stroke Organization: Global Stroke Fact Sheet 2025. Int. J. Stroke.

[2] Gierczyński J., Kułakowska A., Rejdak K. (2024). Neurologia w Polsce. Stan Obecny i Perspektywy Rozwoju.

[3] Feigin V.L., Owolabi M.O. (2023). World Stroke Organization–Lancet Neurology Commission Stroke Collaboration Group. Pragmatic solutions to reduce the global burden of stroke: A World Stroke Organization-Lancet Neurology Commission. Lancet Neurol..

[4] Luchowski P., Rejdak K. (2020). Acute stroke treatment. Lekarz POZ.

[5] Ozdemir H., Sagris D., Abdul-Rahim A.H., Lip G.Y.H., Shantsila E. (2024). Management of ischaemic stroke survivors in primary care setting: The road to holistic care. Intern. Emerg. Med..

[6] Bąk E., Żywczak M., Krzemińska S. (2023). Acceptance of illness and life satisfaction in patients after ichemic stroke. J. Neurol. Neurosurg. Nurs..

[7] Loni E., Sayadnasiri M., Akbarfahimi N., Basakha M., Bidhendi-Yarandi R. (2025). What Is the Optimal Length of Stay for Effective Inpatient Neurorehabilitation? A Retrospective Cohort Study. Health Sci. Rep..

[8] Liao Y.K., Kim T.W., Asami T., Han D.S., Liang H.W. (2026). Post-acute care systems for stroke rehabilitation in Japan, South Korea, and Taiwan. J. Formos. Med. Assoc..

[9] Mazurek K., Blaszkowska A., Rymaszewska J. (2013). Rehabilitacja po udarze mózgu—Aktualne wytyczne. Nowiny Lek..

[10] Siger A. (2024). Sytuacja Osób po Udarze Mózgu w Polsce. Raport 2024.

[11] Kostrzewa A., Kurowska A., Długosz A., Pelczyńska M. (2024). Diagnoza: Udar. Call to action dla Polski i Europy. Forum Zaburzeń Metab..

[12] Canciu A.M., Pintea A.L., Diaconu C., Popa F.L., Domnariu H.P., Domnariu C.D. (2025). The Impact of Post-Stroke Disability on Rehabilitation Costs in Romania. J. Clin. Med..

[13] Folstein M.F., Folstein S.E., McHugh P.R. (1975). Mini-mental state. A practical method for grading the cognitive state of patients for the clinician. J. Psychiatr. Res..

[14] Stańczak J. (2010). MMSE Polish Standardization.

[15] Mahoney F., Barthel D.W. (1965). Functional evaluation: The Barthel index. Md. State Med. J..

[16] Salbach N.M., Mayo N.E., Wood-Dauphinee S., Hanley J.A., Richards C.L., Côté R. (2004). A task-orientated intervention enhances walking distance and speed in the first year post stroke: A randomized controlled trial. Clin. Rehabil..

[17] Hsieh C.L., Sheu C.F., Hsueh I.P., Wang C.H. (2002). Trunk control as an early predictor of comprehensive activities of daily living function in stroke patients. Stroke.

[18] Bohannon R.W. (2006). Reference values for the Timed Up and Go Test: A descriptive meta-analysis. J. Geriatr. Phys. Ther..

[19] Louie D.R., Eng J.J. (2018). Berg Balance Scale score at admission can predict walking suitable for community ambulation at discharge from inpatient stroke rehabilitation. J. Rehabil. Med..

[20] Tomczak M., Tomczak E. (2014). The need to report effect size estimates revisited. An overview of some recommended measures of effect size. Trends Sport Sci..

[21] Lenhard W., Lenhard A. (2022). Computation of Effect Sizes.

[22] O’Dell M.W. (2023). Stroke Rehabilitation and Motor Recovery. Continuum (Minneap Minn).

[23] Belagaje S.R. (2017). Stroke Rehabilitation. Continuum (Minneap Minn).

[24] Rahayu U.B., Wibowo S., Setyopranoto I., Hibatullah Romli M. (2020). Effectiveness of physiotherapy interventions in brain plasticity, balance and functional ability in stroke survivors: A randomized controlled trial. NeuroRehabilitation.

[25] Bijl T., Mudzi W., Comley-White N. (2023). Predictors of patient length of stay post stroke rehabilitation. Afr. Health Sci..

[26] Otokita S., Uematsu H., Kunisawa S., Sasaki N., Fushimi K., Imanaka Y. (2021). Impact of rehabilitation start time on functional outcomes after stroke. J. Rehabil. Med..

[27] He F., Blackberry I., Njovu M., Rutherford D., Mnatzaganian G. (2025). Delayed inpatient rehabilitation and functional outcomes for acute stroke: A retrospective cohort study in an Australian regional hospital. J. Rehabil. Med..

[28] Coleman E.R., Moudgal R., Lang K., Hyacinth H.I., Awosika O.O., Kissela B.M., Feng W. (2017). Early Rehabilitation After Stroke: A Narrative Review. Curr. Atheroscler. Rep..

[29] Bindawas S.M., Vennu V., Mawajdeh H., Alhaidary H.M., Moftah E. (2018). Length of Stay and Functional Outcomes Among Patients with Stroke Discharged from an Inpatient Rehabilitation Facility in Saudi Arabia. Med. Sci. Monit..

[30] Çiftçi B., İkbali Afşar S. (2022). Relationships between lenght of stay for rehabilitation ans functional outcomes in stroke patients. Malang Neurol. J..

[31] James J., McGlinchey M.P. (2022). How active are stroke patients in physiotherapy sessions and is this associated with stroke severity?. Disabil. Rehabil..

[32] Lekander I., Willers C., von Euler M., Lilja M., Sunnerhagen K.S., Pessah R.H., Borgström F. (2017). Relationship between functional disability and costs one and two years post stroke. PLoS ONE.

[33] Angerova Y., Marsalek P., Chmelova I., Gueye T., Uherek S., Briza J., Rogalewicz V. (2020). Cost and cost-effectiveness of early inpatient rehabilitation after stroke varies with initial disability: The Czech Republic perspective. Int. J. Rehabil. Res..

[34] Harini T., Madushani P., Munasinghe D., Maleesha Y., Vithanage K., Wettasinghe A. (2025). The association of cognitive functions with functional outcomes during post-stroke recovery: A cross-sectional study. J. Clin. Neurosci..

[35] Rexrode K.M., Madsen T.E., Yu A.Y.X., Carcel C., Lichtman J.H., Miller E.C. (2022). The Impact of Sex and Gender on Stroke. Circ. Res..

[36] Gall S., Phan H., Madsen T.E., Reeves M., Rist P., Jimenez M., Lichtman J., Dong L., Lisabeth L.D. (2018). Focused Update of Sex Differences in Patient Reported Outcome Measures after Stroke. Stroke.

[37] Guzek Z., Dziubek W., Stefańska M., Kowalska J. (2024). Evaluation of the functional outcome and mobility of patients after stroke depending on their cognitive state. Sci. Rep..

[38] Milosevich E., Kusec A., Pendlebury S.T., Demeyere N. (2025). Domain-specific cognitive impairments, mood and quality of life 6 months after stroke. Disabil. Rehabil..

